# Physiological and molecular responses of seedlings of an upland rice (‘Tung Lu 3’) to total submergence compared to those of a submergence-tolerant lowland rice (‘FR13A’)

**DOI:** 10.1186/s12284-017-0180-3

**Published:** 2017-08-31

**Authors:** Si-Yu Yang, Yu-Sian Wu, Chung-Tse Chen, Ming-Hsin Lai, Hsing-Mu Yen, Chin-Ying Yang

**Affiliations:** 1 Department of Agronomy, National Chung Hsing University, Taichung, 40227 Taiwan; 2 Graduate Institute of Biotechnology, National Chung Hsing University, Taichung, 40227 Taiwan; 3 Crop Science Division, Taiwan Agricultural Research Institute, Taichung, 41362 Taiwan

**Keywords:** Upland rice, Submergence, ROS, Antioxidant enzyme activity, SUS1

## Abstract

**Background:**

Understanding the responses of rice to environmental stresses such as unscheduled submergence is of pressing important owing to increasing severity of weather thought to arise from global climate change. When rice is completely submerged, different types adopt either a quiescence survival strategy (i.e., minimal shoot elongation) or an escape strategy (i.e., enhanced shoot elongation). Each strategy can prolong survival depending on the circumstances. While submergence responses have been studied in rice typical of lowland and flood-prone areas, few studies have explored the physiological and molecular properties of upland rice under submergence. Here, we use seedlings of the upland rice ‘Tung Lu 3’ (‘TL3’) to analyze physiological and molecular responses to submergence. We compare them with those of ‘FR13A’, a lowland rice that tolerates submergence by adopting the quiescence strategy.

**Results:**

Plant height and distance between leaf sheaths, increased rapidly in ‘TL3’ under submergence. Although this indicated a strong escape strategy the seedlings remained totally underwater for the duration of the experiments. In contrast, ‘FR13A’ elongated much less. Consequently, after 4 days complete submergence followed by drainage, ‘TL3’ lodged much more severely than ‘FR13A’. After 10 d complete submergence, 55% of ‘TL3’ seedlings survived compared to 100% in ‘FR13A’. Chlorophyll a, b and total chlorophyll concentrations of the 2^nd^ oldest leaves of ‘TL3’ were also significantly above those of ‘FR13A’ (but were lower than ‘FR13A’ in the 3^rd^ oldest leaves) and less hydrogen peroxide accumulated in ‘TL3’. Peroxidase activity in submerged ‘TL3’ was also greater than in ‘FR13A’ 1 day after submergence. Quantitative RT–PCR showed increased expression of *sucrose synthase 1* and *alcohol dehydrogenases 1* after 2 days complete submergence with significantly higher levels in ‘TL3’ compared to ‘FR13A’. Expression was also higher in ‘TL3’ under non-submerged conditions.

**Conclusions:**

The upland rice line ‘TL3’ gave a stronger elongation response than ‘FR13A’ to complete submergence. This escape strategy is widely considered to prejudice survival when the plant remains totally submerged. However, contrary to expectations, ‘TL3’ survival rates were substantial although below those for ‘FR13A’ while physiological, biochemical and molecular parameters linked to adaptation differed in detail but appeared to be broadly comparable. These findings highlight that submergence tolerance is determine not only by the adoption of quiescence or escape strategies but maybe by metabolic and physiological properties unrelated to the underwater elongation rate.

## Background

The four major ecosystems in rice farming differ in terms of the amount of water required to provide a successful growing environment and can be characterized as follows: (I) the irrigated rice ecosystem. This comprises approximately 55% of the global rice land and produces 75% of the world’s rice; (II) the rainfed lowland rice ecosystem. This comprises approximately 25% of the global rice land and produces 17% of global rice; (III) the upland rice ecosystem. This is typified by dry and infertile soil and makes-up approximately 13% of the global rice land area and produces 4% of global rice; and (IV) the flood-prone rice ecosystem. This is prevalent in South and Southeast Asia especially China, Cambodia, Bangladesh, and Thailand. Flood-prone ecosystems comprise approximately 7% of the global rice land area and produce 4% of global rice. Flood-prone rice is found mainly in river delta areas where deep flooding to 50–400 m is common during the growing season. In this ecosystem, rice is planted before the rainy season and stems later elongate with the rising river water with sufficient vigor to keep the upper shoot above water level (IRRI 1997; Ito et al. 1999).

Increased emissions of carbon dioxide and other ‘greenhouse gases’ such as methane are widely believed to result in global warming and accelerate global climate change (Karl and Trenberth 2003). Increases in global temperatures are causing sea levels to rise, rainfall and snowfall patterns to change and the frequency of extreme weather events to grow. One outcome is the greater prevalence of submergence stress in farming systems that severely depresses crop yields. This applies even to rice because of the severe restrictions submergence imposes for the inward movement of oxygen and carbon dioxide needed for respiration and photosynthesis. The gas transmission rate in water is much slower than in air, resulting in hypoxia and in photosynthetic deficiency (Gibbs and Greenway 2003). Various studies have revealed numerous changes in rice primary metabolism in the absence of oxygen. In particular, several reactions associated with sucrose metabolism and fermentation have been reported (Lakshmanan et al. 2014). Leaf photosynthetic capacity is also constrained under submergence stress because of the shading effect of water. The combined effects hinder leaf formation, reduce the total leaf area, promote leaf senescence, inhibit root growth, reduced tillering in rice and can be fatal (Kato et al. 2014).

‘Escape’ or ‘quiescence’ are two major types of adaptive response to submergence, seen in a wide range of species and based on the vigor of upward shoot elongation initiated by submergence (Ram et al. 2002). In most rice lines, the escape strategy takes the form of accelerated underwater elongation and is most marked in deep water lowland rice where vigorous stem extension prevents total submergence as floodwater gradually rises. However, leaves, stems and coleoptiles of almost all rice types elongate faster in response to submergence provided some oxygen is present. When fast shoot extension is insufficient to allow the plant to re-surface, the expenditure in energy and respirable substrates needed to support the faster underwater growth may threaten survival. The rice line ‘FR13A’ is an exception to this general rule. In contrast to most other rice lines ‘FR13A’ does not elongate faster underwater and thus conserves accumulated respirable biomass thereby adopting the so-called quiescence strategy (Singh et al. 2014). This has been linked to its ability to survive complete submergence for much longer than normal, mediated by energy and substrate conservation. This quiescence strategy is an outcome of insensitivity to the hormone ethylene, which normally promotes shoot elongation in rice (Jackson et al. 1987; Nagai et al. 2010; Perata and Voesenek 2007). The inheritable insensitivity to ethylene is located in the *Sub1* locus of chromosome 9 of ‘FR13A’. It contains genes for Group VII apatala2/ethylene response factors. One of these (*Sub1A-1*) is a mutated form and responsible for suppressing ethylene responsiveness (Xu et al. 2006).

Upland rice possesses several characterstics that contribute to its tolerance of dry conditions (Bernier et al., 2007). For example, the roots of upland rice are thicker, larger, grow deeper and are more laterally spread. Roots of upland rice also have a higher osmolarity than roots of lowland rice and can thus retain moisture more effectively in dry soil (Asch et al. 2005; Ahmadi et al. 2014). Upland rice typically has a larger leaf area, fewer stomata, smaller thick-walled cells and larger vascular bundles compared to lowland rice. Although the term ‘upland rice’ is used widely, however episodes of waterlogging are also common in upland soils. There are many characteristics that vary continuously, with considerable overlap, between lowland and upland cultivars of rice (Chang et al. 1972; Colmer 2003). In contrast, little is known of the physiological and molecular properties of upland rice in relation to submergence stress. To rectify this shortcoming, we describe submergence responses of a typical upland rice line (‘TL3’) and compared them with those of a submergence-tolerant lowland rice (‘FR13A’). We measured elongation, survival, activity of superoxide dismutase (SOD) and other enzymes associated with free radical scavaging, concentrations of total chlorophyll and expression levels of two genes associated with anaerobic metabolism *sucrose synthase 1* (*SUS1*) and *alcohol dehydrogenases 1* (*ADH1*). In this study, our results suggest that the upland rice line ‘TL3’ may exhibit some submergence tolerance, but that it is inferior to ‘FR13A’ in total submergence condition.

## Results

### ‘TL3’ upland rice elongates its seedling shoot more than ‘FR13A’ when submerged but retains a substantial but inferior survival rate

**Fig. 1:**
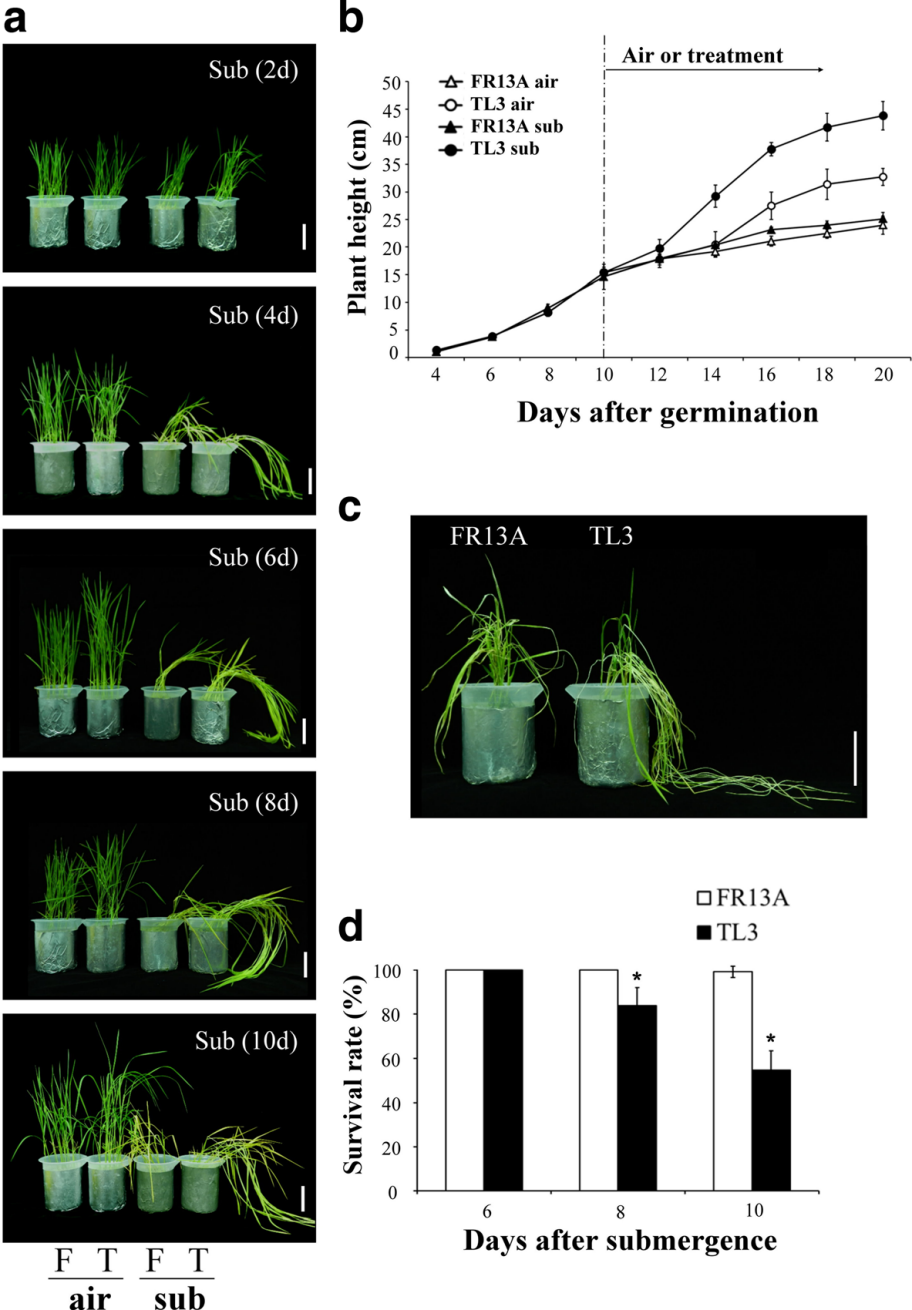
Characterization of ‘TL3’ upland rice and ‘FR13A’ lowland rice seedlings grown under submergence conditions. **a** Photographs of 10-d-old upland and lowland rice seedlings grown in air or submerged for 2, 4, 6, 8, and 10 d. **b** Plant heights of 10-d-old upland and lowland rice seedlings grown in air or submerged for 2, 4, 6, 8, and 10 d. **c** Phenotypes of 10-d-old upland and lowland rice seedlings submerged for 10 d and then allowed to recover for 10 d. **d** Survival rates of seedlings after 6, 8, or 10 d submergence followed by 10 d recovery. Air, non-submerged controls; Sub, full submergence; ‘TL3’, upland rice; and ‘FR13A’, lowland rice. Bar = 7 cm. Data points are means ± standard deviation of three separate experiments each with at least 30 seedlings of each genotype. Asterisks indicate significant differences (*P* < 0.05; Student’s *t*-test)

**Fig. 2:**
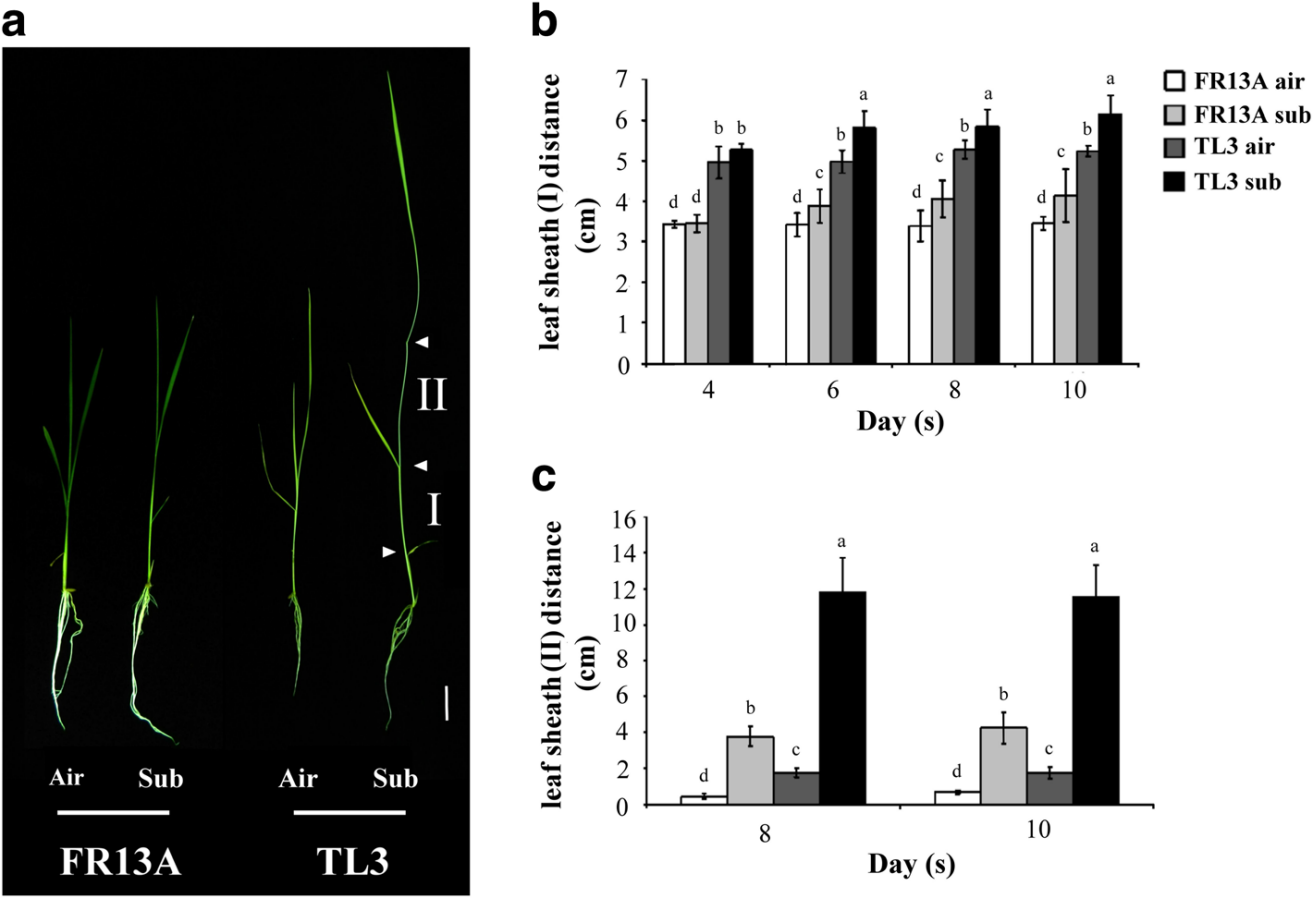
Comparison of leaf sheath lengths in ‘TL3’ and ‘FR13A’ rice seedlings under submergence stress. **a** Photographs of 10-d-old upland and lowland rice seedlings after being exposed to air or submerged for 8 d. The distance from the 1st oldest leaf to the 2nd oldest leaf is leaf sheath distance I, and that from the 2nd oldest leaf to the 3rd oldest leaf is leaf sheath distance II. **b** and **c** Leaf sheath distances I and II of 10-d-old rice seedlings after submergence for 4, 6, 8, and 10 d. Air, non-submerged controls; Sub, full submergence; ‘TL3’, upland rice; and ‘FR13A’, lowland rice. Bar = 3 cm. Data points are means ± standard deviation of three separate experiments each with at least 30 seedlings of each genotype. Values with different letters are significantly different at *P* < 0.05, according to a post-hoc LSD test

Ten-day-old seedlings of ‘TL3’ and ‘FR13A’ were completely submerged for 2, 4, 6, 8, and 10 d and the phenotypic responses measured in terms of elongation, lodging and etiolation (Fig. 1a). After 4 days completely submergence, ‘TL3’ had lodging much more than ‘FR13A’ (Fig. 1a). The average shoot heights of ‘TL3’ were 19.7, 29.2, 37.8, 41.8, and 43.9 cm and those of the ‘FR13A’ were 17.8, 20.4, 27.5, 31.4, and 32.7 cm after complete submergence for 2, 4, 6, 8, and 10 d, respectively (Fig. 1b). Although extension growth by the leaf lamina is a major contributor to overall shoot height (Fig.1). Enhanced elongation by the leaf sheath also contributed to final shoot height of submerged ‘TL3’ (Fig. 2). In ‘TL3’, the length of the leaf sheath distance I (distance between 1^st^ oldest leaf to the 2nd oldest leaf) (Fig.2a) was increased by up to 0.9 cm (Fig. 2b) and that from 2^nd^ oldest leaf to the 3^rd^ oldest leaf (distance II) was increased by up to 10.1 cm (Fig. 2c). Increases in leaf sheath lengths of ‘FR13A’ were very much less than this.

When seedlings were placed under complete submergence for 6, 8, and 10 d and then allowed to recover for 10 d, survival rate was assessed on the basis of an ability to form one or more new leaves. The survival rates of upland rice variety ‘TL3’ after 6, 8, or 10 d submergence were 100%, 84%, and 55%, respectively. Survival rates of the submergence tolerant ‘FR13A’, were, as expected, ~100% for all three submergence periods (Fig. 1c and d). The strongly increasing extension growth by ‘TL3’ during complete submergence is indicative of the escape strategy. But, in the absence of actual escape (the seedlings remained totally submerged during treatment) 55 % of ‘TL3’ seedlings survived even the longest period underwater (10d).

### Submergence decreases leaf chlorophyll concentrations in both ‘TL3’ and ‘FR13A’ seedlings

**Fig. 3:**
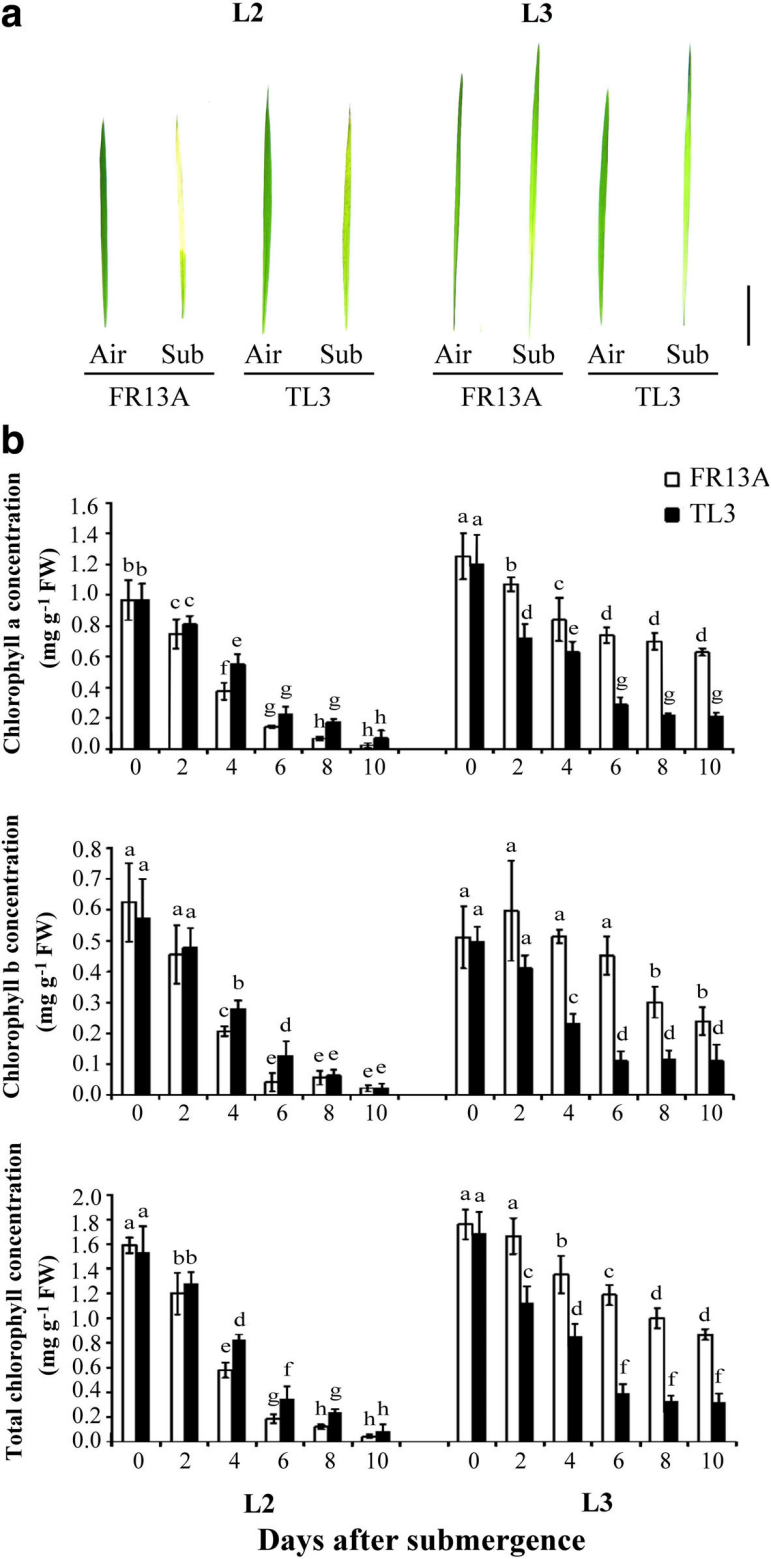
Chlorophyll concentrations in ‘TL3’ and ‘FR13A’ rice seedlings under submerged conditions. **a** Photographs of 10-d-old upland and lowland rice seedlings after being exposed to air (control) or fully submerged (Sub) for 6 d. **b** The a, b and total chlorophyll concentration of 10-d-old seedlings after 0, 2, 4, 6, 8, and 10 d in air (control) or full submergence (Sub). L2, 2^nd^ oldest leaf and L3, 3^rd^ oldest leaf. Bar = 3 cm. Data points are means ± standard deviation of three separate experiments each with at least 30 seedlings of each genotype. Values with the different letters are significantly different (*P* < 0.05; post-hoc LSD test)

Leaf color and chlorophyll concentration of plants under biotic stress (e.g., pathogens, insect pests) or abiotic stress (e.g., high temperature, low temperature, submergence and drought) are similar to those of plants undergoing natural senescence (Matile et al. 1996). To investigate changes in chlorophyll during submergence, 10-d-old ‘TL3’ and ‘FR13A’ seedlings were submerged for 6 d. The 2^nd^ oldest leaves of both ‘FR13A’ and ‘TL3’ became yellow–green but visibly less so in ‘TL3’. The 3^rd^ oldest leaves of both lines yellowed to a similar extent although leaf apices remained green (Fig. 3a). Chlorophyll concentrations in ‘TL3’ and ‘FR13A’ were measured after submergence for 0, 2, 4, 6, 8, and 10 d (Fig. 3b). Chlorophyll a & b and total chlorophyll of the second oldest leaves decreased by about 90% in both lines after 10 d inundation. Concentrations were lower in ‘FR13A’ compared to ‘TL3’. The reverse was true for the third oldest leaves. Here, chlorophyll loss was much stronger in ‘TL3’ throughout the 10d submergence treatment.

### ‘TL3’ accumulates less hydrogen peroxide (H_2_O_2_) during submergence than ‘FR13A’

**Fig. 4:**
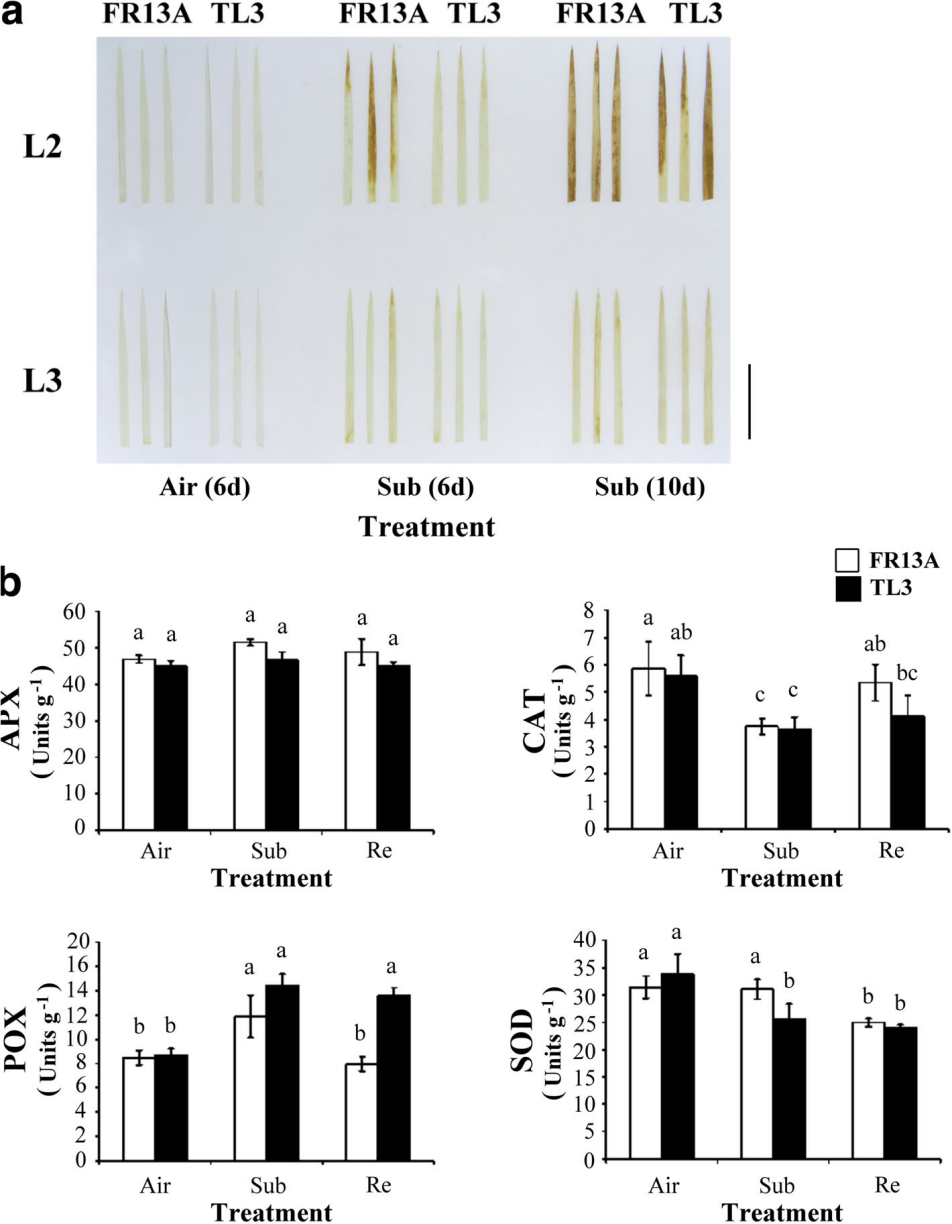
Activities of antioxidative enzymes in ‘TL3’ and ‘FR13A’ rice seedlings under submerged conditions. **a** 3,3′-diaminobenzidine (DAB) staining of H_2_O_2_ in detached 2^nd^ oldest leaves (L2) and 3^rd^ oldest leaves (L3) of 10-d-old rice seedlings after 6 or 10 d in air (control) or full submergence (Sub). Similar results were obtained in three independent experiments. Photographs show the results of three independent leaves. Bar = 3 cm. **b** Enzyme activity of detached 2^nd^ oldest leaves (L2) and 3^rd^ oldest leaves (L3) of 10-d-old rice seedlings after 2d in air (control) or full submergence (Sub) and then allowed to recover for 1 d (Re). Ascorbate peroxidase (APX), catalase (CAT), total peroxidase (POX) and superoxide dismutase (SOD) activities are average values ± standard deviation of four biologically independent experiments each with duplicate samples. Values with the different letters are significantly different at *P* < 0.05, according to a post-hoc LSD test

Nicotinamide adenine dinucleotide phosphate (NADPH) and H^+^ derived from the light reactions of photosynthesis can accumulate in cell membranes generating superoxide anion radicals (O_2_^−^) in the chloroplast thylakoid. These are readily converted by superoxide dismutase (SOD) to H_2_O_2_ and stressed leaves are known to accumulate more H_2_O_2_ than unstressed leaves (Sagi and Fluhr 2006). To determine the amount of H_2_O_2_ accumulated under hypoxic stress, 10-d-old ‘FR13A’ and ‘TL3’ seedlings were submerged for 0, 6, and 10 d and H_2_O_2_ measured by 3,3′-diaminobenzidine staining. Staining of the second oldest leaf 2 was less in ‘TL3’compared to ‘FR13A’ suggesting less H_2_O_2_ in ‘TL3’ (Fig. 4a). No reddish-brown staining was observed in the 3^rd^ leaves of the ‘FR13A’ or ‘TL3’ rice. Cellular scavenging capacity of the antioxidative enzymes, such as catalase (CAT), ascorbate peroxidase (APX), SOD, and total peroxidase (POX) in ‘FR13A’ and ‘TL3’ rice seedlings were then determined after 2d submergence and again after 1 d recovery. Submergence decreased CAT activity and increased POX similarly in both ‘FR13A’and ‘TL3’ (Fig. 4b). There was no effect of submergence on APX. However, SOD activity was significantly depressed by submergence but only in ‘TL3’. After 1 d recovery, POX activity of ‘TL3’ was greater than in that of ‘FR13A’.

### Hypoxia-inducible gene expression levels in submerged ‘TL3’ and ‘FR13A’ seedlings

Previously published studies in rice show hypoxia can increase transcript levels of certain genes such as *SUS1* and *ADH1* that are involved in carbohydrate metabolism (Yang et al. 2011; Yang 2014). To assess the expression of these genes under submergence stress, 10-d-old ‘FR13A’ and ‘TL3’ seedlings were completely submerged for 2 d and transcript levels of *SUS1* and *ADH1* determined by quantitative RT–PCR. Levels of *SUS1* and *ADH1* transcripts increased under complete submergence stress in both ‘FR13A’ and ‘TL3’. Stimulation was equally strong for *ADH1. SUS1* expression was approximately 3-fold higher in non-submerged ‘TL3’ compared to ‘FR13A’ but submergence raised transcripts more in ‘FR13A’ by 84% in ‘TL3’ and 228% in ‘FR13A’. Thus, stimulation of levels of *SUS1* and *ADH1* expression by submergence was substantial in both ‘TL3’ and ‘FR13A’.

## Discussion

Most rice types elongates their shoot more rapidly when submerged and thus adopts the so-called escape strategy for survival. This reaction is seen in the leaves of small seedlings and is successful in promoting survival if, as a result, the shoot regains contact with the aerial environment. Exceptions to the rule are rice lines related to ‘FR13A’. This is a mutated rice that originated in lowland India and was found to be unusually tolerant of complete submergence. The tolerance of FR13A is linked to its ability to conserve respirable reserves by not elongating underwater. This has been termed the quiescence strategy (Karin et al. 1982; Nagai et al. 2010; Perata and Voesenek 2007; Bailey-Serres and Voesenek 2008). However, the position regarding upland rice has not been explored before and we have therefore studied submergence responses of an upland rice cultivar (‘TL3’) and compared it to those of ‘FR13A’. We found that ‘TL3’elongated markedly compared to ‘FR13A’ and lodged severely after desubmergence (Fig. 1a and b). Elongation by the leaf sheath especially that between the second and third oldest leaves (distance II in Fig. 2) contributed to shoot lengthening. We further confirmed the submergence tolerance of ‘FR13’ and showed that although survival of ‘TL3’ is lower than that of ‘FR13A’ after 8–10 d of complete submergence, its survival ability remains substantial (Fig. 1c and d) despite substantial underwater shoot elongation. Therefore, for upland ‘TL3’ rice, the concepts of escape or quiescence strategies do not help understand the considerable resilience of ‘TL3’ to submergence stress (e.g., 84% survival after 8 d complete submergence). Therefore, ‘TL3’ may possess inherent metabolic tolerance to submergence that is probably linked to adaptive changes in its energy metabolism.

It is well-known that chloroplasts disintegrate and photosynthetic capacity is lost when rice is submerged (Mustroph et al. 2010). QTLs have been mapped to increased submergence tolerance through their effects on decreased underwater shoot elongation, to increased levels of chlorophyll and to loci of unknown function (Toojinda et al. 2003). One likely cause is membrane lipid peroxidation by O_2_^−^and H_2_O_2_. The latter is known to accumulate in low oxygen conditions (Santosa et al. 2007; Wu and Yang 2016). Accordingly, leaves of both the upland ‘TL3’ and lowland ‘FR13A’ yellowed when the seedlings were submerged. (Figure 1a). The rate of chlorophyll decline in the 2^nd^ oldest leaves of ‘TL3’ was slower than in ‘FR13A’ but faster in the 3^rd^ oldest leaf (Fig. 3). At the same time, accumulation of H_2_O_2_ in the 2^nd^ oldest leaves of ‘TL3’ was less than in the ‘FR13A’ rice under submergence but greater in the 3^rd^ oldest leaf (Fig. 4) thus linking higher H_2_O_2_ with greater loss of chlorophyll. Although chlorophyll in the 3^rd^ oldest leaves of ‘TL3’ was significant lower than in ‘FR13A’, 80% of ‘TL3’ seedlings survived 8 d submergence (Fig. 1d), thus demonstrating considerable resilience within the limitations of this study in not including a “submergence sensitive” control.

Potentially damaging reactive oxygen species (ROS) are generated more freely in plants under abiotic stress but elevated activities of antioxidant enzymes can reduce the injury and increase stress tolerance (Gill and Tuteja 2010). In our plants, total peroxidase activity (POX) increased in both ‘FR13A’and ‘TL3’ during submergence and this may have lowered H_2_O_2_ sufficiently to contribute to their tolerance of submergence and especially so after submergence when activity in ‘TL3’ was maintained. However, the activity of catalase (CAT), an enzyme that degrades H_2_O_2_ to H_2_O_,_ declined in both ‘TL3’ and ‘FR13A’ during submergence (Fig. 4b) suggesting a minor role in regulating H_2_O_2_ levels. Activity of SOD, the enzyme responsible for converting highly active O_2_^−^ was substantial in both rice lines and was little changed by submersion. Overall, higher H_2_O_2_ is a likely promoter of leaf senescence in submerged ‘TL3’ and ‘FR13A’ but assays of scavanging enzymes did not offer a clear explanation for this or for the different patterns of accumulation between the two lines and between older and younger leaves.

**Fig. 5:**
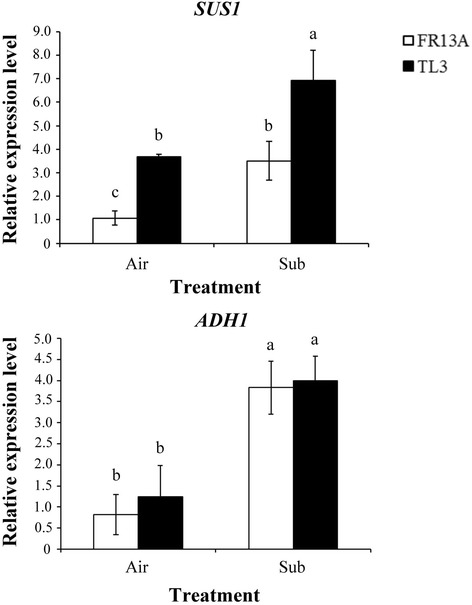
Transcript levels of hypoxia-inducible genes in ‘TL3’ and ‘FR13A’ rice seedlings under submerged conditions. Total RNAs were isolated from shoots of 10-d-old ‘TL3’ upland rice and ‘FR13A’ lowland rice seedlings after 2d in air (control) or full submergence (Sub). Transcript levels of hypoxia-inducible *sucrose synthase 1* (*SUS1*) and *alcohol dehydrogenases 1* (*ADH1*) were determined using quantitative RT–PCR. Relative transcript levels were calculated and normalized to ubiquitin mRNA. Values are means ± standard deviation of three biologically independent experiments each with duplicate samples. Values with the different letters are significantly different at *P* < 0.05, according to a post-hoc LSD test

In non-submerged conditions, plants generate ATP through aerobic respiration of assimilates. Under hypoxic stress, the sucrose synthase (SUS) pathway is upregulated, and invertase activity and gene expression are inhibited. These changes can reduce consumption of ATP used to process sucrose into glycolysis and thus enhance plant survival possibly in conjunction with an enhanced fermentation pathway (Bieniawska et al. 2007). Our quantitative RT–PCR results revealed that *SUS1* expression was increased by submergence in both rice lines, with a proportionately much larger increase in ‘FR13A’. However, in absolute terms, expression in ‘TL3’ was significantly greater than that in ‘FR13A’ regardless of submergence treatment (Fig. 5). Expression of *ADH1* was strongly increased by submergence indicating that fermentation was probably active during submergence. However, the levels of activity were similar in both ‘TL3’ and ‘FR13A’.

## Conclusions

We quantified survival rates and associated changes in plant height and leaf sheath length during submergence of an upland rice (‘TL3’) in comparison with a well-known submergence-tolerant lowland rice (‘FR13A’). The tolerance of ‘FR13A’ was verified and linked to slow rates of underwater leaf and leaf sheath elongation (the quiesence strategy). In contrast, the upland ‘TL3’ elongated rapidly when submerged (the escape strategy). However, even though this extension growth was insufficient to regain contact with the air, survival rates exceeded 50% even after 10 d, suggest a metabolic rather than morphological basis for the tolerance. Submergence promoted chlorosis in both rice lines although older leaves lost more chlorophyll in ‘FR13A’ and younger leaves lost more in ‘TL3’. This pattern was reflected by H_2_O_2_ levels which were higher in the more chlorotic leaves. Additionally, POX activity was raised by submergence but to a similar extent in both lines although this fell-away more in ‘FR13A’ during post-submergence recovery. The hypoxia-related genes *SUS1* and *ADH1* were expressed more strongly under submergence in both rice lines. Overall, the regulatory mechanisms may explain the partial tolerance to submergence, whereby ‘TL3’ elongates in total submergence and thus expending considerable amounts of energy remain unclear and merit further study.

## Methods

### Plant materials and growth conditions

The submergence-tolerant rice variety ‘FR13A’ and upland rice variety ‘Tung Lu 3’ (‘TL3’) were used. Seeds were obtained from Dr. M-H Lai (Taiwan Agricultural Research Institute, Taiwan). Seeds were surface sterilized with 3% sodium hypochlorite for 30 min, washed several times with sterile deionized water and placed on wet filter paper for 3 d for germination in a growth chamber set at 28 °C with 6-h light (236 μmol m^−2^ s^−1^)/ 8-h dark cycle. Germinated seeds were transplanted onto a metal grid over a 500 mL beaker containing Kimura B solution (Yoshida et al. 1976). A total of 40 germinated seeds were placed on the grid of each beaker and the water culture solution changed every 2 d for 10 d (2–3 leaf stage). A total of 30 evenly-sized seedlings from each beaker were selected for subsequent experiments.

### Seedling submergence treatments and survival rate determination

For submergence treatments, 10-d-old seedlings were placed in a water tank (40 cm × 40 cm × 60 cm) filled with 55 cm of water. At this depth, plants could not elongate sufficiently to reach the water surface within 10 d. Seedlings were subjected to complete submergence for 0, 2, 4, 6, 8, and 10 d under a 16-h light/8-h dark cycle. After each treatment, sampled tissues were immediately frozen in liquid nitrogen and stored at −80 °C. The ability to grow new leaves within 7 d of being submerged for 6, 8, and 10 d was taken as a measure of survival. Experiments were repeated three times and at least 30 seedlings were measured independently each time.

### Plant height, leaf sheath length and chlorophyll concentration measurements

After treatment, lengths of each shoot and leaf sheaths of at least 30 plants were recorded at intervals indicated. Chlorophyll a and b were extracted from 50 mg of leaf tissue in 2 mL of sodium phosphate buffer (50 mM pH 6.8), 40 μL of which was added to 960 μL of 99% ethanol and incubated for 30 min at room temperature in the dark with gentle shaking. After centrifugation at 4 °C for 15 min at 1000 g, the absorbance values of the supernatant were measured at 665 and 649 nm with a spectrophotometer (Metertec SP8001).

### Histochemical staining and antioxidative enzyme activity

Accumulation of H_2_O_2_ in cells was visualized by 3,3′-diaminobenzidine staining as previously described (Yang and Hong 2015). The experiments were repeated three times. For the antioxidative enzyme assays, seedlings were first subjected complete submergence for 2 d and then allowed to recover for additional 1 d. Control plants remained unsubmerged. Shoot tissue (50 mg) was excised and immediately used for enzyme extractions. Activity levels of CAT, APX, POX and SOD were analyzed as previously described (Wu and Yang 2016). Each experiment was repeated four times.

### Quantitative RT–PCR analyses

Shoot samples were collected from 10-d-old seedlings and frozen in liquid nitrogen and stored in −80 °C until use. Total RNA was extracted using TRIzol (Invitrogen, Carlsbad, CA, USA) and then subjected to DNase treatment using the TURBO DNA-free Kit (Ambion, Austin, TX, USA). RNA concentrations were determined spectrophotometrically and then reverse transcribed into cDNA using Moloney murine leukemia virus reverse transcriptase (Invitrogen). Quantitative RT–PCR was performed using a Rotor-Gene 3000 instrument (Corbett Research, Sydney, Australia) with Power SYBR Green PCR Master Mix (GeneMark, Taipei, Taiwan) in accordance with the manufacturer’s recommendations. The ubiquitin (*Os03g13170*) gene was used as an internal control to normalize cDNA levels. Relative expression levels were analyzed with Rotor-Gene 6 software (Corbett). Experiments were repeated three times independently with duplicate samples. The sequences of primers used for quantitative RT–PCR are in Table 1.

**Table 1:** Primers used for quantitative RT-PCR experiments

Gene name	Primer sequence
*OsSUS1*- forward	5′-catctcaggctgagactctga −3′
*OsSUS1*- reverse	5′- caaattcaatcgaccttactt −3′
*OsADH1*- forward	5′-gcaaatttctggctttgtcaatcagta −3′
*OsADH1*- reverse	5′-cgccaaaagatcactgattcttaacaa −3′
*Osubiquitin* - forward	5′-aaccagctgaggcccaaga-3’
*Osubiquitin* - reverse	5′-acgattgatttaaccagtccatga-3’

## Abbreviations

APX: Ascorbate peroxidase
CAT: Catalase
H_2_O_2_: Hydrogen peroxide
POX: Total peroxidase
ROS: Reactive oxygen species
RT–PCR: Reverse transcriptase-polymerase chain reaction
SOD: Superoxide dismutase

## References

[CR1] Ahmadi N, Audebert A, Bennett MJ, Bishopp A, de Oliveira AC, Courtois B, Diedhiou A, Dievart A, Gantet P, Ghesquiere A, Guiderdoni E, Henry A, Inukai Y, Kochian L, Laplaze L, Lucas M, Luu DT, Manneh B, Mo XR, Muthurajan R, Perin C, Price A, Robin S, Sentenac H, Sine B, Uga Y, Very AA, Wissuwa M, Wu P, Xu J (2014) The roots of future rice harvests. Rice 7:29 10.1186/s12284-014-0029-y PMC4884021 26224558

[CR2] Asch F, Dingkuhn M, Sow A, Audebert A (2005). Drought-induced changes in rooting patterns and assimilate partitioning between root and shoot in upland rice. Field Crop Res.

[CR3] Bailey-Serres J, Voesenek LACJ (2008). Flooding stress: acclimations and genetic diversity. Annu Rev Plant Biol.

[CR4] Bernier J, Kumar A, Ramaiah V, Spaner D, Atlin G (2007). A large-effect QTL for grain yield under reproductive-stage drought stress in upland rice. Crop Sci.

[CR5] Bieniawska Z, Barratt DHP, Garlick AP, Thole V, Kruger NJ, Martin C, Zrenner R, Smith AM (2007). Analysis of the sucrose synthase gene family in Arabidopsis. Plant J.

[CR6] Chang TT, Loresto GC, Tagumpay O (1972). Agronomic and growth characteristics of upland and lowland rice varieties. Rice breeding.

[CR7] Colmer TD (2003) Aerenchyma and an inducible barrier to radial oxygen loss facilitate root aeration in upland, paddy and deep-water rice (Oryza sativa L.) Ann Bot 91(2):301-309 10.1093/aob/mcf114 PMC4795684 12509350

[CR8] Gibbs J, Greenway H (2003). Mechanisms of anoxia tolerance in plants. I. Growth, survival and anaerobic catabolism (vol 30, pg 1, 1993). Funct Plant Biol.

[CR9] Gill SS, Tuteja N (2010). Reactive oxygen species and antioxidant machinery in abiotic stress tolerance in crop plants. Plant Physiol Biochem.

[CR10] IRRI (International Rice Research Institute) (1997). Rice Almanac.

[CR11] Ito O, Ella E, Kawano N (1999). Physiological basis of submergence tolerance in rainfed lowland rice ecosystem. Field Crop Res.

[CR12] Jackson MB, Waters I, Setter T (1987). Injury to rice plants caused by complete submergence: a contribution by ethylene (ethene). J Exp Bot.

[CR13] Karin S, Vergara BS, Mazaredo AM (1982). Anatomical and morphological studies of rice varieties tolerant of and susceptible to complete submergence at seedling stage. proceedings of the 1981 Intemational Deepwater Rice workshop.

[CR14] Karl TR, Trenberth KE (2003). Modern global climate change. Science.

[CR15] Kato Y, Collard BCY, Septiningsih EM, Ismail AM (2014). Physiological analyses of traits associated with tolerance of long-term partial submergence in rice. AoB PLANTS.

[CR16] Lakshmanan M, Mohanty B, Lim SH, Ha SH, Lee DY (2014). Metabolic and transcriptional regulatory mechanisms underlying the anoxic adaptation of rice coleoptiles. AoB PLANTS.

[CR17] Matile P, Hortensteiner S, Thomas H, Krautler B (1996). Chlorophyll breakdown in senescent leaves. Plant Physiol.

[CR18] Mustroph A, Lee SC, Oosumi T, Zanetti ME, Yang HJ, Ma K, Yaghoubi-Masihi A, Fukao T, Bailey-Serres J (2010). Cross-kingdom comparison of Transcriptomic adjustments to low-oxygen stress highlights conserved and plant-specific responses. Plant Physiol.

[CR19] Nagai K, Hattori Y, Ashikari M (2010). Stunt or elongate? Two opposite strategies by which rice adapts to floods. J Plant Res.

[CR20] Perata P, Voesenek LACJ (2007). Submergence tolerance in rice requires Sub1A, an ethylene-response-factor-like gene. Trends Plant Sci.

[CR21] Ram PC, Singh BB, Singh AK, Ram P, Singh PN, Singh HP, Boamfa I, Harren F, Santosa E, Jackson MB, Setter TL, Reuss J, Wade LJ, Singh VP, Singh RK (2002). Submergence tolerance in rainfed lowland rice: physiological basis and prospects for cultivar improvement through marker-aided breeding. Field Crop Res.

[CR22] Sagi M, Fluhr R (2006). Production of reactive oxygen species by plant NADPH oxidases. Plant Physiol.

[CR23] Santosa IE, Ram PC, Boamfa EI, Laarhoven LJ, Reuss J, Jackson MB, Harren FJ (2007). Patterns of peroxidative ethane emission from submerged rice seedlings indicate that damage from reactive oxygen species takes place during submergence and is not necessarily a post-anoxic phenomenon. Planta.

[CR24] Singh S, Mackill DJ, Ismail AM (2014) Physiological basis of tolerance to complete submergence in rice involves genetic factors in addition to the SUB1 gene. AoB PLANTS 6: plu060 10.1093/aobpla/plu060 PMC4243076 25281725

[CR25] Toojinda T, Siangliw M, Tragoonrung S, Vanavichit A (2003). Molecular genetics of submergence tolerance in rice: QTL analysis of key traits. Ann Bot.

[CR26] Wu YS, Yang CY (2016). Physiological responses and expression profile of NADPH oxidase in Rice (Oryza Sativa) seedlings under different levels of submergence. Rice.

[CR27] Xu K, Xu X, Fukao T, Canlas P, Maghirang-Rodriguez R, Heuer S, Ismail AM, Bailey-Serres J, Ronald PC, Mackill DJ (2006). Sub1A is an ethylene-response-factor-like gene that confers submergence tolerance to rice. Nature.

[CR28] Yang CY (2014). Hydrogen peroxide controls transcriptional responses of ERF73/HRE1 and ADH1 via modulation of ethylene signaling during hypoxic stress. Planta.

[CR29] Yang CY, Hong CP (2015). The NADPH oxidase Rboh D is involved in primary hypoxia signalling and modulates expression of hypoxia-inducible genes under hypoxic stress. Environ Exp Bot.

[CR30] Yang CY, Hsu FC, Li JP, Wang NN, Shih MC (2011). The AP2/ERF transcription factor AtERF73/HRE1 modulates ethylene responses during hypoxia in Arabidopsis. Plant Physiol.

[CR31] Yoshida S, Forno DA, Cock JA, Gomez KA (1976). Laboratory manual for plant physiological studies of Rice, Ed 3.

